# Comparative evaluation of effect of intracanal cryotherapy and corticosteroid solution on post endodontic pain in single visit root canal treatment

**DOI:** 10.4317/jced.61023

**Published:** 2024-03-01

**Authors:** Raji-Viola Solomon, Shanti-Priya Paneeru, Chigurupati Swetha, Rohini Yatham

**Affiliations:** 1 Professor, Department of Conservative Dentistry and Endodontics, Panineeya Institute of Dental Sciences and Research Centre, Hyderabad, Telangana, India; 2 Reader, Department of Conservative Dentistry and Endodontics, Panineeya Institute of Dental Sciences and Research Centre, Hyderabad, Telangana, India; 3 Senior Professor, Department of Conservative Dentistry and Endodontics, Panineeya Institute of Dental Sciences and Research Centre, Hyderabad, Telangana, India; 4 Post graduate student, Department of Conservative Dentistry and Endodontics, Panineeya Institute of Dental Sciences and Research Centre, Hyderabad, Telangana, India

## Abstract

**Background:**

Post-operative pain after endodontic treatment is a common occurrence, and its prevalence is mostly high immediately after treatment, decreasing in one day and reportedly reaching minimal levels 7 days after treatment. This research focuses on the final flushing of the root canal with different irrigating solutions to reduce its occurrence. Aim: To compare and evaluate the effect of delivering three intracanal irrigants - saline at room temperature, saline at 2.5oC, and intracanal dexamethasone as final irrigant on postoperative pain after a single visit endodontics.

**Material and Methods:**

This randomized double-blinded parallel clinical trial was conducted on patients requiring a single appointment root canal treatment, with a pain level of 4 or higher on the visual analogue scale (VAS). According to the final irrigating solution, all patients were randomly allocated into three groups of 15 each: 0.9% physiological saline (Group 1), 2.5oC cold saline (Group 2), and 2ml Dexamethasone (Group 3). Patients were instructed to complete VAS to determine their post-operative scores at 6hrs, 12hrs, and 24hrs following the treatment.

**Results:**

In all groups, mean VAS scores decreased significantly from baseline to 6hr and 12hr, with lower scores at 24 hours. Post-hoc analysis revealed no significant distinction in the mean VAS scores between 12 hours to 24 hours. Lower pain scores are observed in the dexamethasone group, followed by cryotherapy which performed better than saline at room temperature.

**Conclusions:**

Final flushing with dexamethasone solution and cryotherapy is effective, safe, and a well-tolerated strategy to minimize postoperative pain after single visit endodontics.

** Key words:**Intracanal cryotherapy, Cold saline, Dexamethasone, Postoperative pain, Visual analogue scale.

## Introduction

Endodontic therapy eliminates the pulpal and peri-radicular disease-causing agents and improves tissue recovery. Further, with the advent of contemporary root canal treatment protocols and current innovative rotary file systems, newer irrigating materials and techniques, novel intracanal medicaments, recent root canal sealers, and improved obturating materials and techniques, the incidence of post-operative endodontic pain has reduced considerably. However, its prevalence is almost always unavoidable because it depends on several variables including the patient’s characteristics, complex root canal anatomy, pulpal and periapical status, and microbial factors (1).

Unpleasant postoperative pain has a high-frequency rate of around 3 to 58% and tends to worsen between the initial 6 and 12 hours after treatment, peaking at around 40% in 24 hours, and then declining to 11% in one week (2). Periapical tissue irritation is most likely one of the primary causes of postoperative endodontic discomfort. It results in nociceptor activation and local inflammatory reactions, which cause the inflamed and injured tissues to release chemical mediators such as prostaglandins (PGs), bradykinin, and cytokines, ultimately leading to peripheral sensitization and a higher incidence of traumatic pain (3).

Given the multi-factorial aetiology of post-operative endodontic pain, several measures have been suggested to reduce its incidence. Non-steroidal anti-inflammatory medications (NSAIDs) are one of the most frequently used methods to bring down the incidence of postoperative pain, which acts by competitive inhibition of the cyclooxygenase (COX) enzyme. A meta-analysis by Smith *et al*. (4) concluded that NSAIDs are effective in reducing postoperative pain. However, a systematic review and network meta-analysis by Zanjir *et al*. (5) observed only low to moderate effectiveness of NSAIDs in patients with reversible pulpitis.

Alternatively, glucocorticoids also show promise and scope in reducing post-operative pain, which acts by inhibiting the synthesis and release of arachidonic acid and its metabolites, thereby inhibiting the synaptic pain pathways. Dexamethasone is a synthetic form of the glucocorticoid class that has been used through submucosal and supra-periosteal injections to reduce post-treatment swelling and pain following endodontic treatment (6-7). An exhaustive study and meta-analysis conducted by Nath *et al*. and co-workers have proven that corticosteroids have a substantial effect on the reduction of postoperative pain at 4-6 hours and 12 hours after endodontic treatment (8). Patients who received corticosteroids before Inferior Alveolar Nerve Block were 70.7% more likely to experience no pain or moderate discomfort than patients in the control group, although the difference was not statistically significant after 24 hours. Side effects of this approach were low to non-existent.

Cryotherapy, also known as cold therapy, is a more specific procedure effective in lowering tissue temperature, vascular blood flow, and nerve impulse conduction velocity, which in turn lessens pain, inflammation, edema, and recovery time.(9) The first clinical investigation on the use of intracanal cryotherapy in pain control was undertaken by Al-Nahlawi *et al*., (10) using cold saline (2-40C), and observed considerable pain reduction. At the time, of its inception cryotherapy was largely applied to intraoral surgical operations to reduce the intensity and post-surgical occurrence. Later, a few studies evaluated its effectiveness in decreasing post-endodontic discomfort (11-14). However, there is limited substantial evidence on the standardization of time, duration, application method, and type of cold agent employed, and the current research focus is on regulating its manner of use and operating guidelines.

The unique area of interest in this current research investigation was to evaluate the use of simple, cost-effective biocompatible solutions to achieve a reduction in the pain score levels after single-visit root canal procedures in symptomatic teeth. This could be of promise as a viable alternative to the use of NSAIDs to manage post-operative pain, especially in geriatric individuals and patients with allergic immunological responses. Hence to compare and evaluate the effect of delivering three different intracanal irrigants - saline at room temperature, 2.50c cold saline, and dexamethasone as the final irrigant on postoperative pain after a single visit root canal treatment.

## Material and Methods

A Randomized, double-blinded parallel clinical trial was carried out at the Department of Conservative Dentistry and Endodontics, Panineeya Mahavidyalaya Institute of Dental Sciences and Research Centre in Hyderabad. The Institutional Ethics Review Board granted the ethical clearance for the study and allotted the number PMVIDS& RC/IEC/ENDO/PR/543-22. The consent form was signed by subjects who volunteered to participate, and after that, the study procedure and protocols were informed to them along with their merits and limitations. The participation was voluntary, and the respondents’ identities and confidentiality were protected. The study was compiled according to CONSORT Guidelines and conducted following the Helsinki Declaration.

The sample size was estimated using the G power software (Version 3.1.9.6, Franz Faul, Universitat Kiel, Germany) based on the previous literature analysis, the minimum sample size obtained for the current study design was 45 (with 15 study participants in each intracanal irrigant group). As the study requires a recall follow-up schedule, to overcome a possible attrition rate, a total sample size of 50 was taken into consideration.

Patients in the age group of 18-40 years with symptomatic irreversible pulpitis with either normal apical tissues or symptomatic apical periodontitis in single rooted teeth with single root canal requiring a single appointment root canal treatment, with a pain level of 4 or higher on the visual analogue scale, who gave their informed written consent were undertaken for the study. Patients with a compromised contributing medical history, with a recent history of antibiotic or analgesic usage to control the pain, and teeth with multiple canal complexities and shape, primary endodontic failures/re-treatments, root resorption pathologies, open apex, and periapical pathology were excluded from the study.

Participants were given a visual analogue scale (VAS) with a scale from 0-10 and asked to rate their level of pain. The patients were instructed and trained in the process of evaluating their pain using the VAS scale. After analysing the history of the present illness, clinical and radiographic examination, and results of the cold and electric pulp testing the diagnosis was made and an appropriate treatment plan was formulated to carry out a single-visit endodontic procedure. The same endodontist performed the therapy in one visit to reduce bias and adhere to the same standard operating protocols throughout the procedure.

Preparation of Subjects: An endodontic access cavity preparation was made utilizing an endo access bur after the injection of local anaesthesia (2% lignocaine with adrenaline 1:80,000 adrenaline) and rubber dam isolation. With #2-3 Gates-Glidden drills, the coronal portion of each canal was flared, and pulp extirpated with a barbed broach. An ISO K-file # 10 was passed through the canal up to the apical foramen and checked for canal patency. The working lengths were determined using a Root ZX mini electronic apex locator (Morita Corp., Kyoto, Japan) and confirmed radiographically using Radio Visio Graphy. The root canals were instrumented with a ProTaper Next (Dentsply Tulsa Dental Specialties, Johnson City, TN, USA) system upto F3 after performing apical calibration. The initial apical binding file was determined and final enlargement upto 3 sizes larger was maintained for each tooth. Chemo mechanical debridement was done under copious irrigation with 5ml of 17% EDTA followed by 5.25% NaOCl.

According to the final irrigating solution to be used in the canal, all subjects were randomly allocated into three groups of 15 each. The random allocation of study subjects in each group was performed using the lottery method. The procedure of Group allocation and concealment was carried out independently by a separate investigator. Both the participant and the investigator were blinded.

**Figure 1:**
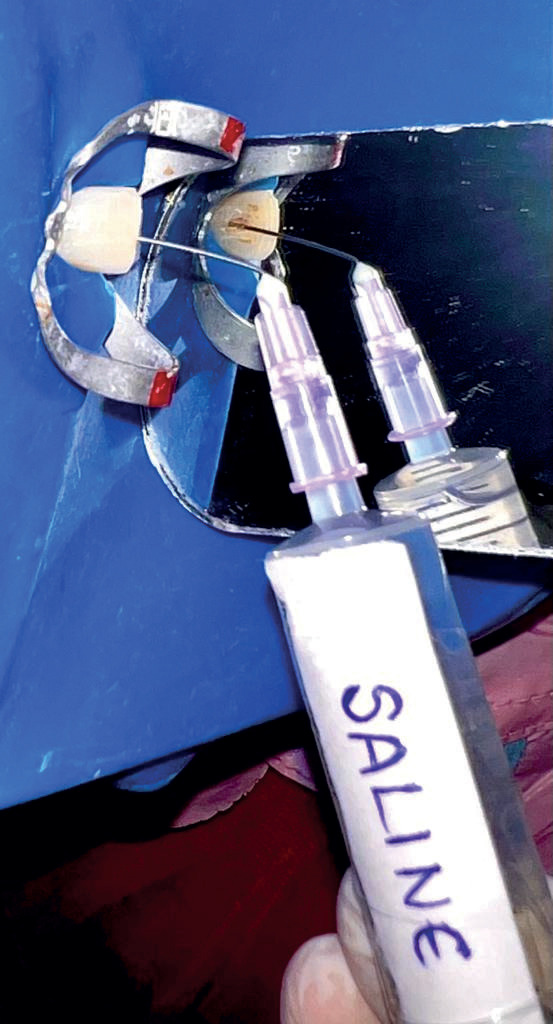
Represents the final irrigation in Group 1 – control group (room temperature saline).

**Figure 2:**
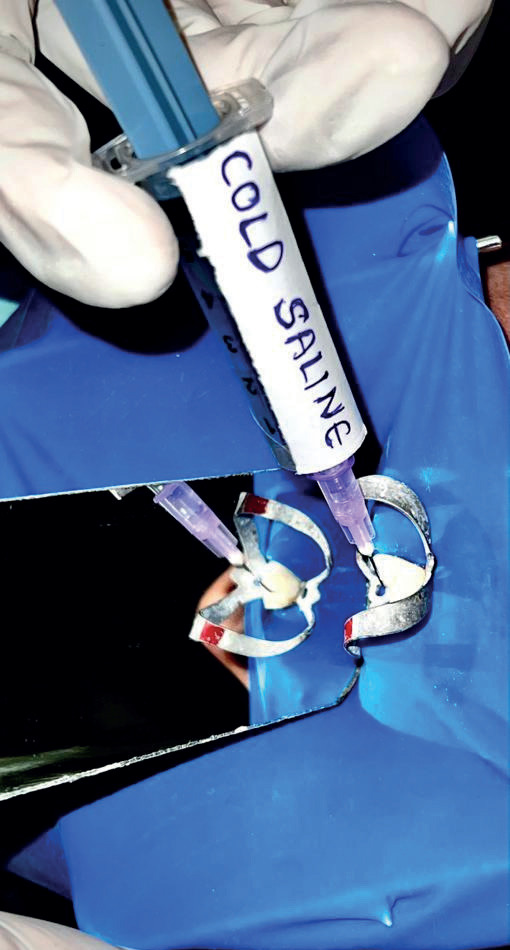
Represents the final irrigation in Group 2 – cold saline.

**Figure 3:**
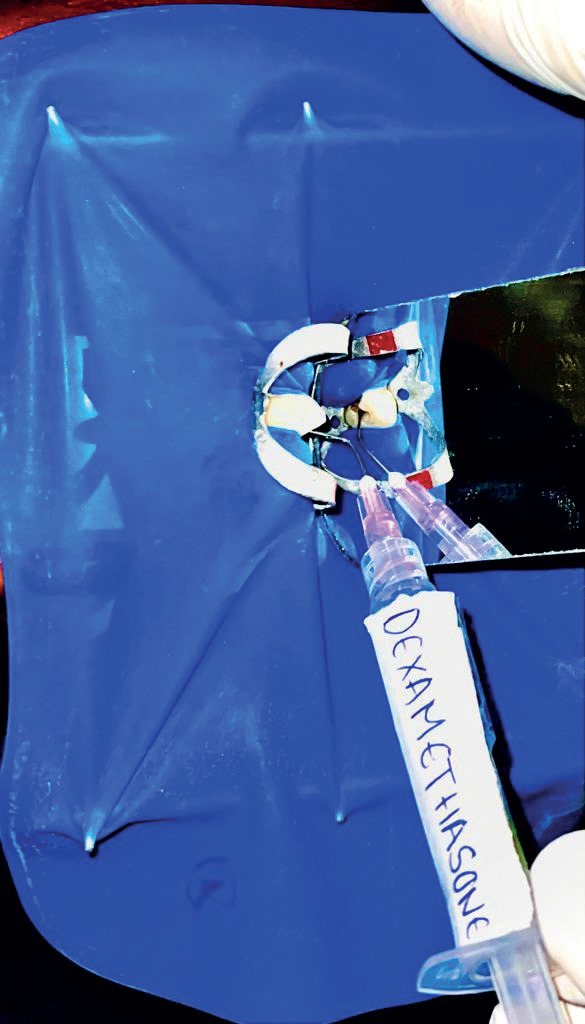
Represents the final irrigation in Group 3 – dexamethasone.

Subjects in each group received 5ml of 0.9% of physiological saline (Group 1-control group Fig. 1), 5 ml of 2.50C cold saline (Group 2 Fig. 2), and 2ml of Dexamethasone (Group 3 Fig. 3). The prepared canals were irrigated with the above irrigating solutions with a 31-gauge side vented needle for five minutes and dried with paper points, AH sealer was applied and then obturated with single cone obturation technique and coronal seal with composite resin restoration.

Once the coronal seal was evaluated and the occlusal check performed the rubber dam was removed and post-operative instructions were given. The patients from each of the respective groups underwent similar treatment protocols by the same endodontist and single visit therapy was followed. After the procedure, all the subjects were reminded and motivated to complete VAS to determine their post-operative scores at 6hrs, 12hrs, and 24hrs.

-Statistical analysis: The data was evaluated using the Statistical Package for Social Sciences (SPSS) version 21(IBM, Armonk, NY, USA). The normality of the sample was assessed using the Shapiro-Wilk test and it was observed to be a uniform distribution, thus parametric tests were applied. A comparison of pain reduction between the timelines in each group (within-group comparison) was conducted using Repeated Measures of ANOVA. While comparison of mean pain reduction between the three groups at each timeline was done using One-Way ANOVA. The Chi-square test and Mc Nemar test were used to compare the number of subjects free of pain. The value *p*≤ 0.05 was considered statistically significant.

## Results

Table 1 signifies the mean comparison of VAS scores within each group at different timelines. Among the subjects that received the normal physiological saline, the mean VAS scores decreased significantly (*p*=0.04) from baseline to (6.73± 1.27) to each follow-up, with lower scores at 24 hours (0.86±1.40). In group 2 (cold saline), a similar reduction of mean VAS scores was seen and is statistically significant (6.86± 1.30 to 0.66±1.29; *p*=0.00). In group 3, the mean VAS scores decreased significantly (*p*=0.0) from baseline to (7.26± 1.22) each follow-up, with lower scores at 24 hours (0.53±1.12). Post-hoc analysis revealed that among all three groups, there was no significant distinction in the mean VAS scores between 12 hours to 24 hours (*p*=0.324, 0.115, 1.000 respectively).

**Table 1:**
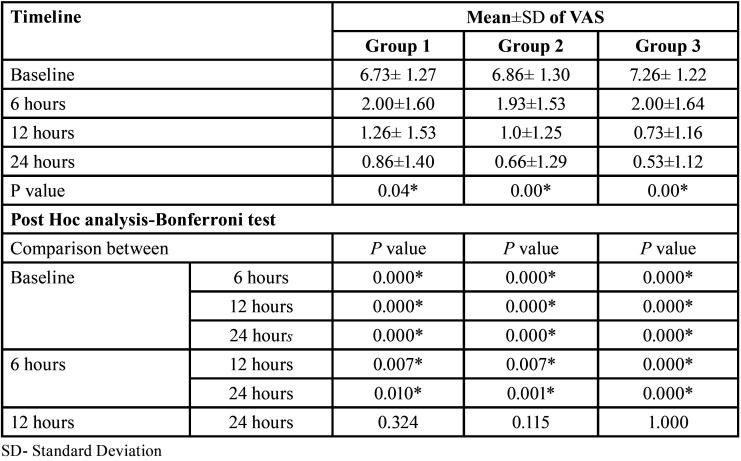
Mean comparison of VAS scores between each timeline within each group.

Table 2 represents the mean comparison of VAS scores between each group at each follow-up. At baseline, the mean VAS scores were comparable between each group with no significant difference (*p*=0.186). At 6hr follow-up, the teeth that were irrigated with cold saline had comparably lower mean VAS scores (1.93±1.53), while similar scores were seen when normal saline and dexamethasone. Nonetheless, the comparison was not statistically significant (*p*=0.102). However, at 12 and 24 hours the VAS scores were significantly lower when dexamethasone was used as an irrigant (0.73±1.16, 0.53±1.12 respectively) and higher with normal saline (1.26± 1.53, 0.86±1.40 respectively).

**Table 2:**
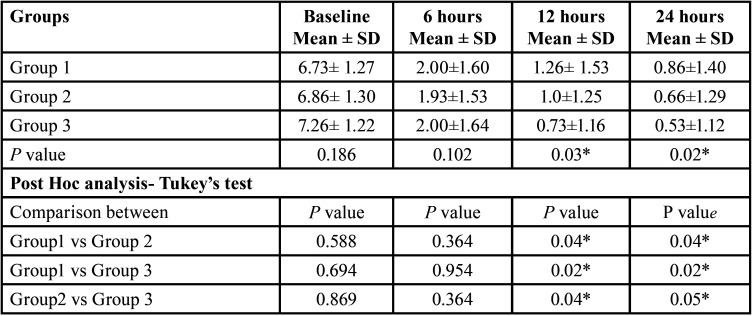
Mean comparison of VAS scores between groups at each follow-up.

Table 3 represents the comparison of pain status between the groups at each timeline. It was observed that at each follow-up, there was no significant variation concerning the pain status between the groups (*p*=0.879, 0.701, 0.897 respectively). At 6-hour follow-up, subjects in group 1 were comparably lower in number for the presence of pain (73.3%) in comparison to other groups (80%). Nonetheless, at 12 hours of follow-up, comparably, a greater percentage of subjects were pain-free when dexamethasone was given as an irrigant (60%). During at 24-hour follow-up, both the cold saline group and the dexamethasone group had more subjects who reported no pain (73.3%) in comparison to the normal saline group (66.7%).

**Table 3:**
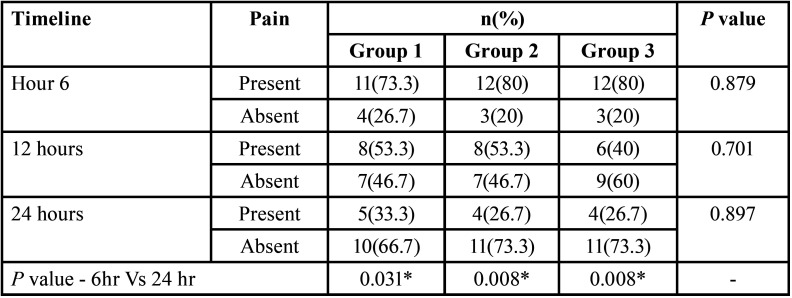
Distribution based on the pain status at each timeline.

## Discussion

In most cases of necrotic or infective pulps, an environment favourable for harbouring a myriad of oral bacterial species and microbial biofilm colonies is created. The incidence of inflammation associated with postoperative pain could be reduced by adopting an appropriate root canal disinfection protocol that eradicates almost all the components of the dentinal smear layer and tenacious bacterial biofilms that reside within the gap of the radicular canal walls as well as inside its anatomical complexities.

Conventional control of pain with preoperative medication using NSAIDs, opioids, or steroids administered either orally or systemically has shown the potential to lessen post-operative pain levels. However, it may lead to numerous gastrointestinal and systemic adverse effects, especially with prolonged usage. An alternative technique that has been proposed is the intracanal administration of a long-lasting analgesic and anti-inflammatory drug, which helps in the sustained release of the drug over gradual periods, thereby creating an anti-bacterial effect and promoting optimal healing.

Dexamethasone, a corticosteroid with potent anti-inflammatory activity, helps to block phospholipase-A2, which in turn lowers the production of prostaglandins and leukotrienes, prevents polymorphonuclear leukocyte chemotaxis and hinders the release of free radicals. In addition, numerous proinflammatory cytokines involved in the inflammatory process of the disease and host immunological response can be downregulated (15).

The literature (6,7) reveals that various experimental and clinical studies have shown it to be effective before, mid-treatment, and after the treatment procedure and that it can be administered via multiple routes. The intracanal administration of Dexamethasone may be regarded as safe because it can be used in lower doses as it easily diffuses from the canal onto the surrounding tooth root’s apex and periapical tissues enabling it to be held in contact for a longer period with the inflamed tissues for better action. Taking this into account the present randomized double-blinded parallel clinical trial was designed to use 2ml of Dexamethasone as the final intracanal irrigant to assess its efficacy in reducing post-operative pain at the 6hr, 12hr, and 24hr periods.

The use of cryotherapy in Endodontics is a relatively recent method of care that allows solutions of low temperatures to be delivered either locally or systemically to facilitate repair and other therapeutic benefits. It is a relatively simple, cost-effective, safe, and beneficial technique for reducing post-operative pain. An initial rapid decline in the local temperature leads to decreased cellular metabolism, which results in cells using less oxygen, and reduced blood flow due to vasoconstriction, which has the anti-edema effect that subsequently lowers inflammation (16).

Certain studies found no plausible relationship between the frequency of flare-ups and the number of visits (17), whereas others recommended single-treatment visits for a lower incidence of flare-ups and favourable outcomes (18). Contrarily, other research investigations have shown that flare-ups are more frequent with single-visit root canal therapy (19). So, postoperative pain in single-visit endodontic treatment was examined.

The most common occurrence of postoperative pain appears within the first 24 hours of obturation, usually subsides within a few hours, and rarely ever lasts for several days. Thus, any strategy used for pain reduction should be most effective during the first 24 hours. Hence in the present study subjects in both groups were evaluated for pain reduction at 6,12, and 24 hours respectively. The intensity of pain was evaluated using the Visual Analog Scale (VAS) it evaluates the degree of pain that a patient experiences, which can range from none to extreme (20).

In the present study, in the group that received normal saline as an irrigant, there was a significant reduction in pain from baseline to 24 hours. Though there was a reduction in pain from 12 hours to 24 hours, the mean difference was not statistically significant. This finding might be due to the ability of normal saline to flush and get rid of the remnants of NaOCl irrigation, which is responsible for causing irritation and post-endodontic pain. A similar reduction in pain from baseline to 24-48 hours postoperative was reported by Alharthi *et al*. (12) Jaiswal *et al*. (13), Jain *et al*. (14), and Kumari N *et al*. (21). However, Al-Nahlawi *et al*. (10) and Abdullah *et al*. (22) observed the highest values of pain after 6 and 12 hours of treatment respectively, which reduced during monitoring periods, and almost diminished after one week. This difference might be due to the difference in preparation technique or mode of delivery of irrigant.

It is believed that by reducing the tissue nociceptors’ activation threshold and the conduction velocity of pain, cold saline irrigation produces a local anaesthetic effect. Vera *et al*. in their *in vitro* study (23) highlighted the fact that the cold saline application decreased the root’s temperature to an unknown extent, which extended to the periapical region, potentially exerting a local anti-inflammatory effect by reducing edema. In line with the present study findings, Cryotherapy is effective in reducing postoperative pain at 6hrs (10,11,14,24), 24hrs (10,11,14,25,26), 48hrs (10,25,26), and 72hrs (11,24,27).

Dexamethasone works by suppressing the inflammatory mediators, which prevents the development of increased vascular permeability and tissue fluid buildup, hence lowering tissue pressure and discomfort.(6) Intracanal irrigation of dexamethasone after endodontic treatment proved to be effective in lessening pain in the first 12 hours. This rapid reduction in pain might be due to the direct and immediate delivery of the drug to the inflamed periapical tissues. The literature supports that the dosage and the mode of delivery do not influence the anti-inflammatory effectiveness of Dexamethasone (15,28-29).

Between-group comparison of postoperative pain revealed lower pain levels in the dexamethasone group and cryotherapy group with a significant difference only after 12 and 24 hours of treatment. As the literature search did not reveal any study that compared the intracanal irrigation of cold saline with dexamethasone, findings could not be compared. Regardless of the agent, volume, or temperature employed, the cryotherapy groups in the majority of the trials had lower postoperative pain scores when compared to the control groups, even though the protocols between the studies varied greatly (10-12,25,27,30). In contrast, few studies (13,21,26) did not observe any significant difference in pain reduction between normal and cold saline.

The percentage of subjects reporting pain decreased significantly across all groups from 100% to 70-80% in the first six hours, 40-50% at 12 hours, and 20-30% by 24 hours when compared to baseline values. However, there were no significant differences in pain relief between normal saline, cold saline, and dexamethasone at any time, which is consistent with the findings of Vera *et al*. (11) and Jaiswal *et al*. (13) 

## Conclusions

Within the limitations of the present study, we can conclude that a significant reduction in the mean pain scores from baseline to 12 hours was evidenced in all three test groups. A comparison of pain scores between the groups at the different time intervals revealed lower pain scores with the dexamethasone group, followed by cold saline which performed better than saline at room temperature.
